# Effect of intraoperative blood transfusion on inflammatory response in parturients with placenta previa undergoing cesarean section: A prospective observational study

**DOI:** 10.1016/j.heliyon.2023.e13375

**Published:** 2023-02-02

**Authors:** Ji-Hoon Sim, Hyun-Seok Cho, Dong-Min Jang, Hee-Sun Park, Woo-Jong Choi, Jong Yeon Park

**Affiliations:** Department of Anesthesiology and Pain Medicine, Asan Medical Center, University of Ulsan College of Medicine, Seoul, Republic of Korea

**Keywords:** Inflammatory response, Neutrophil to lymphocyte ratio, Obstetric, Transfusion

## Abstract

**Background:**

The neutrophil to lymphocyte ratio (NLR), platelet to lymphocyte ratio (PLR), and red cell distribution width (RDW) have been reported as useful biomarkers for evaluating inflammation and a predictor of surgical prognosis. Although there have been recent reports that transfusion may affect inflammatory responses, studies on the post-transfusion inflammatory response in parturients are rare. Therefore, this study aimed to observe changes in inflammatory response after transfusion during cesarean section (C-sec) through NLR, PLR, and RDW.

**Methods:**

Parturients aged 20–50 years who underwent C-sec under general anesthesia due to placenta previa totalis from March 4, 2021, to June 10, 2021 were participated in this prospective observational study. We compared postoperative NLR, PLR, and RDW between the transfusion and non-transfusion groups.

**Results:**

A total of 53 parturients were included in this study, of which 31 parturients received intraoperative transfusions during C-sec. There were no significant difference in preoperative NLR (3.6 vs. 3.4, p = 0.780), PLR (132.8 vs. 111.3, p = 0.108), and RDW (14.2 vs. 13.6, p = 0.062) between the two groups. However, postoperative NLR was significantly higher in the transfusion group than in the non-transfusion group (12.2 vs. 6.8, p < 0.001). Postoperative RDW was significantly higher in the transfusion group than in the non-transfusion group (14.6 vs. 13.9, p = 0.002) whereas postoperative PLR was not significantly different between the two groups (108.0 vs. 117.4, p = 0.885).

**Conclusions:**

Postoperative NLR and RDW, the inflammatory biomarkers, were significantly higher in the transfused C-sec parturients. These results suggest a significant association between postoperative inflammatory response and transfusion in obstetric practice.

## 1 Introduction

In obstetric care, the incidence of blood transfusion is reported to be 0.2–3.2% [1], and up to 68.8% for parturients with placenta previa [2] which is a major risk factor for obstetric hemorrhage and massive transfusions during maternal cesarean section (C-sec) [2]. Although transfusion is one of the important life-saving interventions in parturients at high bleeding risk, it has been reported to induce an inflammatory condition [3]: The inflammatory response caused by blood transfusion manifests as a systemic inflammatory response syndrome that can lead to severe complications such as sepsis, or multiple organ failure [4].

The neutrophil to lymphocyte ratio (NLR), platelet to lymphocyte ratio (PLR), and red cell distribution width (RDW) are simple and objective biomarkers measured in peripheral blood [5]. These indicators reflect inflammatory status and overall immune system function [6], and are associated with patient survival and surgical prognosis in various diseases such as several cancers [7]. In obstetrics, inflammatory markers such as NLR increase in patients with diseases such as pre-eclampsia [8], hyperemesis gravidarum [9], and gestational diabetes [10], and may be significantly associated with intima-media thickness of common carotid artery in healthy pregnant women [11]. A recent study reported that NLR correlates with transfusion volume and is closely associated with early mortality in trauma patients who underwent massive transfusions for severe bleeding [12]: This study suggests that blood transfusion may increase postoperative inflammatory markers, which may be associated with poor surgical prognosis. However, to our knowledge, there were no studies on post-transfusion inflammatory responses in parturients who underwent C-sec.

Therefore, this study aimed to investigate inflammatory response following blood transfusion through NLR, PLR, and RDW, which are inflammatory biomarkers, in parturients with placenta previa totalis (PPT) who underwent C-sec.

## 2 Materials and methods

### 2.1 Study design

This prospective observational study was approved by the Institutional Review Board of Asan Medical Center (Protocol number: 2019–1090) and was conducted from December 2019 to June 2021 at the Asan Medical Center. This study was conducted in accordance with the Declaration of Helsinki. Parturients who underwent C-sec under general anesthesia due to PPT from March 4, 2021, to June 10, 2021 were assessed for eligibility. The informed consent of each parturient was obtained prior to inclusion in the study.

Parturients aged 20–50years were enrolled, and the following parturients were excluded from the study: (1) parturients aged <20 or ≥50 years; (2) parturients who suffering severe illness (American Society of Anesthesiology physical status ≥3); (3) parturients who underwent blood transfusion before surgery; (4) parturients who refused of transfusion; and (5) parturients who refused to participate in this study.

### 2.2 Anesthetic technique

After essential surgical monitoring (noninvasive blood pressure measurement, pulse oximetry, electrocardiography, and capnography), general anesthesia by intravenous bolus injection of propofol (2–2.5 mg/kg) and succinylcholine (1.5–2.0 mg/kg) was induced. After endotracheal intubation, parturients were mechanically ventilated using sevoflurane 2–4 vol% at a tidal volume of 6–8 mL/kg, and an end-tidal carbon dioxide partial pressure was maintained at 35–40 mmHg. Invasive arterial monitoring was routinely conductedand a central venous catheter was inserted if placental adhesions were suspected. Crystalloid solutions (plasma solution or Ringer's lactate solution) or colloid solutions (5% albumin, synthetic colloid (Voluven®; Fresenius Kabi, Bad Homburg, Germany) were administered during anesthesia. Intraoperative hemoglobin (Hb) < 8 g/dL were transfused with packed red blood cells (RBCs). RBCs transfusion units were determined based on the degree of surgical bleeding. As judged by the anesthesiologist, phenylephrine or ephedrine were administered in situations where the mean arterial blood pressure <65 mmHg.

### 2.3 Data collection & outcome evaluation

All parturients data were obtained, including demographic variables, perioperative data, and laboratory tests at pre- and postoperative day (POD) 0. The demographic variables included age, height, weight, body mass index (BMI), gestational age, parity numbers, gestational diabetes mellitus (DM), gestational hypertension (HTN), preoperative steroid use, previous C-sec and myomectomy history, and placenta accreta/increta. Parturients' laboratory tests were determined within 2 days before surgery in the ward, and at postoperative day (POD 0) for all parturients. Laboratory tests of preoperative, and POD 0 included white blood cell, hemoglobin, platelet count, INR,creatinine, total neutrophil count, total lymphocyte count, NLR, PLR, and RDW. NLR and PLR were calculated as the ratio between absolute neutrophil count to absolute lymphocyte count and between absolute platelet count to absolute lymphocyte count, respectively. Intraoperative data included operation time, crystalloid, colloid, urine output, estimated blood loss, and transfusion. Estimated blood loss was calculated through visual estimates of surgical blood loss, suctioned blood volume measurement, and blood-soaked gauze visual analogue. Postoperative variables included the use of analgesics, such as intravenous fentanyl. The primary aim was the comparison of postoperative NLR between the non-transfusion and transfusion groups. The secondary aim was the comparison of postoperative PLR and RDW between the non-transfusion and transfusion groups.

### 2.4 Statistical analyses

Based on a previous pilot data in our center, we calculated the required sample size by referring to the postoperative NLR; the average value and standard deviation of the postoperative NLR were 8.66 ± 4.06 in the non-transfusion group. The difference of the average values of postoperative NLR between the non-transfusion group and transfusion group was assumed to be approximately 40%; thus, the postoperative NLR was assumed to be 12.12 ± 4.06 in the transfusion group. In the two-sided test with a significance level of 0.05, when the desired power of 80% and the allocation ratio is 1:1, the required number of samples was about 22 in each group. Adjusting for a 20% of dropout rate, a total of 28 patients were considered as ideal for a statistical power.

The independent *t*-test or Mann-Whitney *U* test was used to compare quantitative variables, and a chi-squared or Fisher exact test was performed to analyze qualitative data. Quantitative data are reported as median with interquartile range or mean ± standard deviation, and qualitative data are presented as frequencies and percentages. Data were analyzed with IBM SPSS ver. 22 (IBM Corp., Armonk, NY) and P value less than 0.05 was considered statistically significant.

## 3 Results

**Fig. 1:**
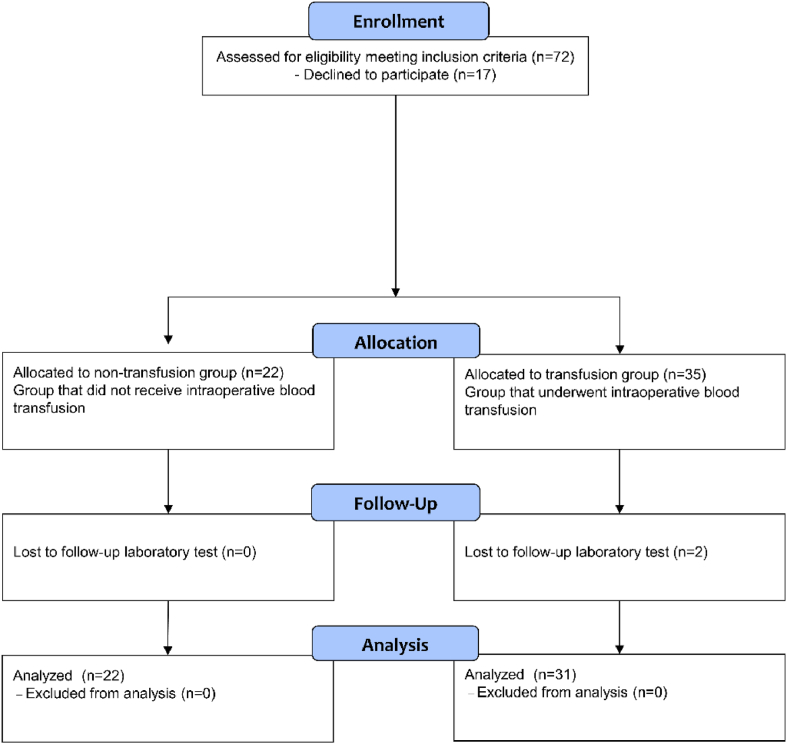
The study flow chart.

A total of 72 parturients were screened for eligibility and 17 parturients declined to participate in this study. Therefore, a total of 55 parturients were included in this study, of which 33 parturients received intraoperative transfusions during C-sec. However, two parturients in the transfusion group were excluded because they did not perform postoperative follow up laboratory test. Finally, a total of 22 parturients in the non-transfusion group and 31 parturients in the transfusion group were analyzed (Fig. 1).

Table 1 shows the demographic characteristics of each group among non-transfusion and transfusion parturients. There were significant differences in weight (p = 0.015), placenta accreta/increta (p = 0.001), and hemoglobin (p = 0.002). By contrast, there were no significant differences in age (p = 0.724), height (p = 0.680), BMI (p = 0.056), gestational age (p = 0.193), parity numbers (p = 0.279), gestational DM (p = 1.000), gestational HTN (p = 0.505), preoperative steroid use (p = 0.132), previous C-sec (p = 0.370), previous myomectomy (p = 1.000), preoperative white blood cell (p = 0.823), platelet count (p = 0.119), INR (p = 0.622), creatinine (p = 0.582), absolute neutrophil count (p = 0.864), absolute lymphocyte count (p = 0.723), NLR (p = 0.780), PLR (p = 0.108), and RDW (p = 0.062) between the two groups.

**Table 1:** Baseline characteristics of the study population.

	Non-transfusion group (n = 22)	Transfusion group (n = 31)	*P*
Age (year)	36.3 ± 4.7	35.9 ± 3.2	0.724
Weight (kg)	68.0 (64.0–72.0)	62.0 (57.3–68.1)	0.015
Height (cm)	160.4 ± 5.3	159.7 ± 6.4	0.680
BMI (kg/m^2^)	27.1 ± 3.6	25.3 ± 3.2	0.056
Gestational age (days)	266.0 (262.0–270.0)	264.0 (258.0–268.0)	0.193
Parity numbers			0.279
0	11 (50.0%)	12 (38.7%)	
1	10 (45.5%)	13 (41.9%)	
≥2	1 (4.5%)	6 (19.4%)	
Gestational DM	2 (9.1%)	2 (6.5%)	1.000
Gestational HTN	0 (0.0%)	2 (6.5%)	0.505
Steroid use	0 (0.0%)	4 (12.9%)	0.132
Previous cesarean section	4 (18.2%)	9 (29.0%)	0.370
Previous myomectomy	1 (4.5%)	2 (6.5%)	1.000
Placenta accreta/increta	0 (0.0%)/0 (0.0%)	9 (29.0%)/5 (16.1%)	0.001
WBC count (1000/μL)	8.0 ± 1.8	7.9 ± 1.8	0.823
Hemoglobin (g/dl)	12.3 (11.7–12.8)	11.3 (10.7–12.0)	0.002
Platelet count (1000/μL)	193.5 ± 47.6	215.7 ± 52.2	0.119
INR	0.97 (0.95–0.99)	0.97 (0.95–0.98)	0.622
Creatinine (mg/dL)	0.51 (0.45–0.55)	0.51 (0.45–0.61)	0.582
Absolute neutrophil count (1000/μL)	5.7 ± 0.4	5.6 ± 0.6	0.864
Absolute lymphocyte count (1000/μL)	1.7 ± 0.5	1.7 ± 0.5	0.723
Preoperative NLR	3.4 (2.8–4.3)	3.6 (2.8–4.5)	0.780
Preoperative PLR	111.3 (96.2–135.1)	132.8 (104.8–166.3)	0.108
Preoperative RDW	13.6 (13.0–14.2)	14.2 (13.4–14.9)	0.062

Data are expressed as mean (standard deviation), number (%), or median (interquartile range). BMI, body mass index; DM, diabetes mellitus; HTN, hypertension; WBC, white blood cells; INR, international normalized ratio; NLR, neutrophil to lymphocyte ratio; PLR, platelet to lymphocyte ratio; RDW, red cell distribution width.

The intraoperative and postoperative variables of each group are listed in Table 2. There were no significant differences in operation time (p = 0.067), total infused crystalloid (p = 0.850), colloid use (p = 0.172), urine output (p = 0.926), and analgesic use (p = 0.391).

**Table 2:** Intraoperative, postoperative variables and study outcomes.

	Non-transfusion group (n = 22)	Transfusion group (n = 31)	*P*
Intraoperative variables			
Operation time (min)	46 (33.0–60.4)	58 (44.0–72.5)	0.067
Crystalloid (mL)	2000.0 (1800.0–2300.0)	1950.0 (1462.5–2375.0)	0.850
Colloid use	4 (18.1)	11 (32.3)	0.172
Urine output (mL)	50 (45.0–90.0)	60 (40.0–100.0)	0.926
Estimated blood loss (mL)	1000.0 (1000.0–1100.0)	1500.0 (1000.0–2375.0)	0.007
Transfusion			
RBC, units	0 (0.0–0.0)	2.4 (2.0–5.8)	<0.001
Massive transfusion (≥10 units)	0 (0.0)	4 (12.9)	0.132
FFP	0 (0.0)	11 (35.5)	0.001
PC	0 (0.0)	2 (6.5)	0.505
**Postoperative variables**			
Analgesic use	16 (72.7%)	19 (61.3%)	0.391
**Study outcomes**			
Postoperative NLR	6.8 (5.0–9.4)	12.2 (8.0–14.4)	<0.001
Postoperative PLR	117.4 (90.4–161.3)	108.0 (90.4–172.9)	0.885
Postoperative RDW	13.9 (13.2–14.3)	14.6 (13.9–14.9)	0.002

Data are expressed as mean (standard deviation), number (%), or median (interquartile range). RBC, red blood cell; FFP, fresh frozen plasma; PC, platelet concentrates; NLR, neutrophil to lymphocyte ratio; PLR, platelet to lymphocyte ratio; RDW, red cell distribution width.

In the transfusion group, the administered median RBC was 2.4 units, 4 parturients (12.9%) underwent massive transfusion (≥10 units) and FFP and PC were administered to 11 (32.3%) and 2 (6.5%) parturients, respectively.

The median value of NLR at POD 0 were 6.8 in the non-transfusion group and 12.2 in the transfusion group. The NLR at POD 0 was significantly higher in the transfusion group than in the non-transfusion group (p < 0.001). The median value of PLR and RDW at POD 0 were 117.4 and 13.9 in the non-transfusion group and 108.0 and 14.6 in the transfusion group, respectively. The RDW at POD 0 was significantly higher in the transfusion group than in the non-transfusion group (p = 0.002), whereas the PLR was not significantly different between the two groups (p = 0.885).

## 4 Discussion

Our study showed that the postoperative NLR and RDW, inflammatory biomarkers, were significantly higher in the transfused C/sec parturients. These results indicate a significant association between postoperative inflammatory response and blood transfusion in obstetric care.

Previous studies have reported that NLR as a biomarker for pregnancy-related complications. NLR was reported to be significantly higher in gestational diabetes [13], pre-eclampsia [8], and acute pancreatitis in pregnancy [14]. NLR has been reported to better reflect placental inflammatory status than serum C-reactive protein (CRP). High NLR predicted impending preterm birth in the context of normal CRP levels [15]. Recently, a study reported that NLR may be a predictive marker for detection of postoperative infection during C/sec [16]. In addition, postoperative NLR has been reported as a significant risk factor of postoperative complications in other conditions [17]. However, to date, there is no study on the effect of intraoperative transfusion on postoperative inflammatory response in parturients with placenta previa. Our study is first study to evaluate the relationship between the postoperative inflammatory biomarkers and intraoperative transfusion in parturients with PPT who undergoing C-sec.

In this study, the mean preoperative NLR in parturients was 3.6, which is higher than the mean NLR (1.65) in the Korean general population [18], and was consistent with the mean NLR (3.5) of pregnant women in the third trimester of Korea [19]. In our study, the higher postoperative NLR and RDW increases in the transfusion group compared to the non-transfusion group strongly suggest that intraoperative transfusion may increase the postoperative inflammatory response. Previous studies have reported that transfusion increases the inflammatory response in vivo [20], and vitro [21]. Although this transfusion-induced inflammatory response is not fully understood, it can be explained by several mechanisms:

First, when transfusion is associated with side effects such as febrile non-hemolytic transfusion reactions, allergy, hypotension, it is mostly associated with alloimmune immunity, which can lead to severe inflammatory conditions [3,22]. Alloimmunization refers to an immune reaction to foreign antigens from another human, most commonly occurring after blood transfusions, this is due to collisions between the high-affinity receptors found on the recipient's cells and the ligands brought on by the transfused components [23]. Second, leukocytes transported with blood were considered to be a major cause of inflammatory response. Residual leukocytes are very potent immunizers and can affect overall immunization score [24]. Therefore, leukoreduction targeting less than 106 residual leukocytes per blood component after filtration is strongly recommended by the European Community and the American Association of Blood Banks [25]. Third, aging of erythrocytes, extravascular hemolysis, and the subsequent significant increases in circulating iron, is associated with inflammatory response in experimental models in previous studies [26]. Recent studies showed that transfusion is responsible for inflammatory response, although there is no clear relationship with the storage duration [27]. Fourth, infection due to the transfer of pathogenic substances such as bacterial contamination, acute parasitic infection, or viral infection is one of the most feared complications of transfusions [28].

In this study, we found no significant difference in postoperative PLR between the transfusion and non-transfusion groups. This partially supports the existing previous argument that PLR may not be recommended as a prognostic indicator [29].

Our study has some limitations. First, we confirmed the relationship between transfusion and inflammatory responses such as NLR, an inflammatory marker, and was not designed to investigate the relationship between transfusion or increased inflammatory marker and surgical prognosis. Therefore, additional well-designed studies are needed on the relationship between increased inflammatory biomarkers and postoperative complications due to transfusion. Second, in this study, an experiment was performed using blood products prepared for transfusion received from a hospital's blood bank. Fresh, untreated blood is not readily administered for blood transfusion and was therefore not considered relevant in this study. Third, only the laboratory test values on preoperative and POD 0 were used in our analysis. Thus, well-designed prospective studies with long-term laboratory values are needed.

## 5 Conclusions

In conclusion, intraoperative transfusions may increase postoperative NLR and RDW in parturients with PPT receiving C-sec. These results strongly suggest a link between intraoperative transfusion and inflammatory response and show that, although blood transfusion is a life-saving treatment, it may increase the postoperative inflammatory response and adversely affect the patient's prognosis. Therefore, it is considered that appropriate perioperative blood transfusion therapy is needed in parturients with PPT who undergoing C-sec. Further well-designed studies are needed to investigate the relationship between transfusions, increased inflammatory response and surgical prognosis.

## Funding statement

This research was supported by Research Grant funded by a Korean Society of Obstetric Anesthesiologists (grant number: 2019–01).

## Author contribution statement

Ji-Hoon Sim, Hyun-Seok Cho, Woo-Jong Choi: Conceived and designed the experiments; Performed the experiments; Analyzed and interpreted the data; Wrote the paper.

Dong-Min Jang, Hee-Sun Park: Performed the experiments.

Jong Yeon Park: Analyzed and interpreted the data; Contributed reagents, materials, analysis tools or data.

## Ethics approval and consent to participate

The institutional review board of Asan Medical Center (protocol no. 2019–1090). The informed consent was obtained from each participant before inclusion.

## Availability of data and materials

The dataset used and/or analyzed during the current study is available from the corresponding author upon reasonable request.

## Declaration of competing interest

The authors declare that they have no competing interests.

## References

[1] Mhyre J.M., Shilkrut A., Kuklina E.V., Callaghan W.M., Creanga A.A., Kaminsky S. (2013). Massive blood transfusion during hospitalization for delivery in New York State, 1998-2007. Obstet. Gynecol..

[2] Spiegelman J., Mourad M., Melka S., Gupta S., Lam-Rachlin J., Rebarber A. (2017). Risk factors for blood transfusion in patients undergoing high-order Cesarean delivery. Transfusion.

[3] Garraud O., Tariket S., Sut C., Haddad A., Aloui C., Chakroun T. (2016). Transfusion as an inflammation hit: knowns and unknowns. Front. Immunol..

[4] Gajic O., Rana R., Winters J.L., Yilmaz M., Mendez J.L., Rickman O.B. (2007). Transfusion-related acute lung injury in the critically ill: prospective nested case-control study. Am. J. Respir. Crit. Care Med..

[5] Li H., Lu X., Xiong R., Wang S. (2017). High neutrophil-to-lymphocyte ratio predicts cardiovascular mortality in chronic hemodialysis patients. Mediat. Inflamm..

[6] Song M., Graubard B.I., Rabkin C.S., Engels E.A. (2021). Neutrophil-to-lymphocyte ratio and mortality in the United States general population. Sci. Rep..

[7] Templeton A.J., McNamara M.G., Šeruga B., Vera-Badillo F.E., Aneja P., Ocaña A. (2014). Prognostic role of neutrophil-to-lymphocyte ratio in solid tumors: a systematic review and meta-analysis. J. Natl. Cancer Inst..

[8] Serin S., Avcı F., Ercan O., Köstü B., Bakacak M., Kıran H. (2016). Is neutrophil/lymphocyte ratio a useful marker to predict the severity of pre-eclampsia?. Pregnancy Hypert..

[9] Caglayan E.K., Engin-Ustun Y., Gocmen A.Y., Sari N., Seckin L., Kara M. (2016). Is there any relationship between serum sirtuin-1 level and neutrophil-lymphocyte ratio in hyperemesis gravidarum?. J. Perinat. Med..

[10] Hessami K., Tabrizi R., Homayoon N., Hashemi A., Heydari S.T., Pourhoseini S.A. (2021). Gestational diabetes mellitus and inflammatory biomarkers of neutrophil-lymphocyte ratio and platelet-lymphocyte ratio: a systematic review and meta-analysis. Biomarkers : biochemical indicators of exposure, response, and susceptibility to chemicals. Biomarkers.

[11] Sonaglioni A., Esposito V., Caruso C., Nicolosi G.L., Bianchi S., Lombardo M. (2020). Association between neutrophil to lymphocyte ratio and carotid artery wall thickness in healthy pregnant women. Eur. J. Obstet. Gynecol. Reprod. Biol..

[12] Duchesne J.C., Tatum D., Jones G., Davis B., Robledo R., DeMoya M. (2017). Multi-institutional analysis of neutrophil-to-lymphocyte ratio (NLR) in patients with severe hemorrhage: a new mortality predictor value. J. Trauma Acute Care Surgery.

[13] Yilmaz H., Celik H.T., Namuslu M., Inan O., Onaran Y., Karakurt F. (2014). Benefits of the neutrophil-to-lymphocyte ratio for the prediction of gestational diabetes mellitus in pregnant women. Exp. Clin. Endocrinol. Diabetes: Offic. J. German Society Endocrinol. German Diabetes Associat..

[14] İlhan M., İlhan G., Gök A.F., Bademler S., Verit Atmaca F., Ertekin C. (2016). Evaluation of neutrophil-lymphocyte ratio, platelet-lymphocyte ratio and red blood cell distribution width-platelet ratio as early predictor of acute pancreatitis in pregnancy. J. Matern. Fetal Neonatal Med. : the official J. European Associat. Perinatal Med. Federat. Asia Oceania Perinatal Societ. Int. Society Perinatal Obstet.

[15] Kim M.A., Lee Y.S., Seo K. (2014). Assessment of predictive markers for placental inflammatory response in preterm births. PLoS One.

[16] Rotem R., Erenberg M., Rottenstreich M., Segal D., Yohay Z., Idan I. (2020). Early prediction of post cesarean section infection using simple hematological biomarkers: a case control study. Eur. J. Obstet. Gynecol. Reprod. Biol..

[17] Wang Y., Hu X., Su M.C., Wang Y.W., Che G.W. (2020). Postoperative elevations of neutrophil-to-lymphocyte and platelet-to-lymphocyte ratios predict postoperative pulmonary complications in non-small cell lung cancer patients: a retrospective cohort study. Curr. Med. Sci.

[18] Lee J.S., Kim N.Y., Na S.H., Youn Y.H., Shin C.S. (2018). Reference values of neutrophil-lymphocyte ratio, lymphocyte-monocyte ratio, platelet-lymphocyte ratio, and mean platelet volume in healthy adults in South Korea. Medicine.

[19] Hershko Klement A., Hadi E., Asali A., Shavit T., Wiser A., Haikin E. (2018). Neutrophils to lymphocytes ratio and platelets to lymphocytes ratio in pregnancy: a population study. PLoS One.

[20] Hod E.A. (2015). Red blood cell transfusion-induced inflammation: myth or reality. ISBT Sci. Ser..

[21] Urner M., Herrmann I.K., Buddeberg F., Schuppli C., Roth Z'graggen B., Hasler M. (2012). Effects of blood products on inflammatory response in endothelial cells in vitro. PLoS One.

[22] Martí-Carvajal A.J., Solà I., González L.E., Leon de Gonzalez G., Rodriguez-Malagon N. (2010). Pharmacological interventions for the prevention of allergic and febrile non-haemolytic transfusion reactions. Cochrane Database Syst. Rev..

[23] Alves V.M., Martins P.R., Soares S., Araújo G., Schmidt L.C., Costa S.S. (2012). Alloimmunization screening after transfusion of red blood cells in a prospective study. Rev. Bras. Hematol. Hemoter..

[24] Pavenski K., Freedman J., Semple J.W. (2012). HLA alloimmunization against platelet transfusions: pathophysiology, significance, prevention and management. Tissue Antigens.

[25] Sharma R.R., Marwaha N. (2010). Leukoreduced blood components: advantages and strategies for its implementation in developing countries. Asian J. Transfus. Sci..

[26] Hod E.A., Godbey E.A. (2016). The outsider adverse event in transfusion: Inflammation. Presse. Med..

[27] Stark M.J., Keir A.K., Andersen C.C. (2013). Does non-transferrin bound iron contribute to transfusion related immune-modulation in preterms?. Arch. Dis. Child. Fetal Neonatal Ed..

[28] Shander A., Lobel G.P., Javidroozi M. (2016). Transfusion practices and infectious risks. Expet Rev. Hematol..

[29] Lee Y.H., Song G.G. (2018). Neutrophil-to-lymphocyte ratio, mean platelet volume and platelet-to-lymphocyte ratio in Behçet's disease and their correlation with disease activity: a meta-analysis. Int. J. Rheumat. Dis..

